# Evaluation of seven common lipid associated loci in a large Indian sib pair study

**DOI:** 10.1186/1476-511X-11-155

**Published:** 2012-11-14

**Authors:** Sajjad Rafiq, Kranthi Kumar M Venkata, Vipin Gupta, DG Vinay, Charles J Spurgeon, Smitha Parameshwaran, Sandeep N Madana, Sanjay Kinra, Liza Bowen, Nicholas J Timpson, George Davey Smith, Frank Dudbridge, Dorairaj Prabhakaran, Yoav Ben-Shlomo, K Srinath Reddy, Shah Ebrahim, Giriraj R Chandak

**Affiliations:** 1 Faculty of Epidemiology and Population Health, London School of Hygiene and Tropical Medicine, London, UK; 2 Centre for Cellular and Molecular Biology (CCMB), Council of Scientific and Industrial Research (CSIR), Habshiguda, Uppal Road, Hyderabad, 500007, India; 3 South Asia Network for Chronic Disease, Public Health Foundation of India, C-1/52, Safdarjung Development Area, New Delhi, 110016, India; 4 School of Social and Community Medicine, University of Bristol, Bristol, UK; 5 MRC Centre for Causal Analyses in Translational Epidemiology, University of Bristol, Bristol, UK; 6 Bloomsbury Centre for Genetic Epidemiology and Statistics, London, UK; 7 Centre for Chronic Disease Control, New Delhi, India; 8 Public Health Foundation of India, New Delhi, India; 9 Genetic Epidemiology and Bioinformatics Research Group, Human Genetics Research Division, University of Southampton, School of Medicine, Southampton General Hospital, Hants, UK

**Keywords:** SNP, Fulker’s Association model and Lipid traits

## Abstract

**Background:**

Genome wide association studies (GWAS), mostly in Europeans have identified several common variants as associated with key lipid traits. Replication of these genetic effects in South Asian populations is important since it would suggest wider relevance for these findings. Given the rising prevalence of metabolic disorders and heart disease in the Indian sub-continent, these studies could be of future clinical relevance.

**Methods:**

We studied seven common variants associated with a variety of lipid traits in previous GWASs. The study sample comprised of 3178 sib-pairs recruited as participants for the Indian Migration Study (IMS). Associations with various lipid parameters and quantitative traits were analyzed using the Fulker genetic association model.

**Results:**

We replicated five of the 7 main effect associations with p-values ranging from 0.03 to 1.97x10^-7^. We identified particularly strong association signals at rs662799 in *APOA5* (beta=0.18 s.d, p=1.97 x 10^-7^), rs10503669 in *LPL* (beta =−0.18 s.d, p=1.0 x 10^-4^) and rs780094 in GCKR (beta=0.11 s.d, p=0.001) loci in relation to triglycerides. In addition, the *GCKR* variant was also associated with total cholesterol (beta=0.11 s.d, p=3.9x10^-4^). We also replicated the association of rs562338 in *APOB* (p=0.03) and rs4775041 in *LIPC* (p=0.007) with LDL-cholesterol and HDL-cholesterol respectively.

**Conclusions:**

We report associations of five loci with various lipid traits with the effect size consistent with the same reported in Europeans. These results indicate an overlap of genetic effects pertaining to lipid traits across the European and Indian populations.

## Background

Cholesterol and triglycerides are major plasma lipids and key heritable risk factors for cardiovascular disease [1,2]. Common genetic variations, environmental influences and interaction effects between them can alter circulating levels of plasma lipids. Genome wide association studies (GWASs) have been instrumental in identification of several single nucleotide polymorphisms (SNPs), which are associated with altered plasma lipid levels. Evidence for a role of these SNPs as mediators of variation in plasma lipid levels in non-Europeans would demonstrate wider relevance of GWAS findings. This could enhance the possibility of extending potential clinical benefits such as identification of novel therapeutic agents and risk prediction beyond populations in which GWASs were initially performed [3]. More realistically such studies could identify novel associations at the known loci, due to variation in linkage disequilibrium structure.

A recent genome wide meta-analysis under the Global lipid genetics consortium, comprising over 100,000 European individuals identified 95 loci as associated with at least one of the three main lipid traits including low-density lipoprotein cholesterol (LDL-Cholesterol), high-density lipoprotein cholesterol (HDL-Cholesterol) and triglycerides (TG) in individuals of Caucasian descent [4]. Follow up analysis in 9,705 South Asians residing in the UK (LOLIPOP-London Life Sciences Prospective Population Study) identified a majority of SNP associations to be in the same direction as reported in the Caucasian samples, yet a majority of the initial associations failed to replicate in the South Asian (4). Association studies are needed to replicate and establish the true effect sizes of established variants for lipid traits in indigenous South Asian populations. It is known that the prevalence of metabolic syndrome in South Asians residing in urban locations and developed countries [5,6] is higher when compared to individuals from rural areas [7]. As such, association studies conducted across the urban and rural Diasporas within the sub-continent can generate more accurate estimates of genetic effect sizes. In addition, the Indian population is an amalgamation of distinct endogamous genetic sub groups ^9^. The average population differentiation within 19 Indian sub populations was identified to be 3 fold greater than that observed in a study of 23 European population groups [8]. Hence, it is important to ensure that precautions are taken to prevent false positive associations due to population substructure, while conducting genetic association studies in the South Asian populations.

The primary analysis in this study aimed to replicate associations of eight common genetic variants which are known to be associated with key lipid traits in European populations, in an indigenous Indian involving 3178 sib-pairs from the Indian Migration Study (IMS). We studied the effect of variants in *APOA5, APOB*, *APOE/CI/C4*, *CETP*, *LDLR*, *GCKR*, *LPL*, and *LIPC* on lipid traits which included triglyceride levels, LDL-cholesterol, HDL-cholesterol and total cholesterol (TC) levels. All the SNPs that were selected for the present study have been identified to be associated with lipid traits at genome wide significance in previous GWASs [4,9,10]. The lipid trait associated with any of the studied loci at the highest GWAS level of significance in European studies was termed as the primary lipid trait for those loci. Subsequent analysis also included investigation of the association of loci with secondary lipid traits which may or may not be associated with the studied variants in previous European studies. We also investigated the role of gene-environment effects in influencing the primary trait associations.

## Results

A total of 6356 individuals from 3178 sibling pairs were included in the analysis. Rural dwellers were younger, more likely to be male and had a better cardio-metabolic profile (Table 1). We successfully genotyped eight SNPs arising from distinct loci which are known to be associated with key lipid traits including total cholesterol, LDL-cholesterol, HDL-cholesterol, and triglycerides, and were also known to carry rare mutations associated with Mendelian abnormalities of lipid metabolism. Minor allele frequencies for the eight SNPs ranged from 7-21% and call rates were greater than 95%. Duplicate error rates were less than 3%. One SNP rs4420638 at the *APOE/C1/C4* locus failed HWE in the Hyderabad samples (p=3 x 10^-4^) and was excluded from analysis.

**Table 1:** **Characteristics of the sib**-**pairs from the Indian Migration Study**

**Study traits**	**Indian Migration Study **(**IMS**)			
	**Urban dwellers**	**Rural dwellers**	**P**-**value**	**All participants**
**Sample Size**	4032	2324		6356
**Men** (%)	52.6%	66.3%	<0.0001^§^	57.6%
**Age** (**Years**)	41.5 (9.4)^*^	39.9 (11.5)	<0.0001^||^	40.8 (10.3)
**Body Mass Index **(**kg**/**m**^**2**^)	24.9 (4.4)	22.0 (4.1)	<0.0001^||^	23.4 (1.21)
**Triglycerides**^‡^	137.5 (71.3)	128.2 (64.6)	<0.0001^||^	134.1 (69.1)
**Total Cholesterol**^‡^	186.5 (44.8)	177.5 (44.9)	<0.0001^||^	183.2 (45.1)
**HDL**-**cholesterol**^‡^	43.8 (9.4)	43.0 (9.4)	0.003	43.5 (9.5)
**LDL**-**cholesterol**^‡^	114.8 (39.1)	110.5 (69.6)	<0.0001^||^	113.2 (52.4)
**Hypertension**^†^	18.8%	16.9%	<0.0001^§^	18.1%

Data presented as mean (SD); ^*^SD: Standard deviation; ^†^Hypertension was defined using cut-off of Systolic Blood Pressure ≥140 mm of Hg and Diastolic Blood Pressure ≥90 mm of Hg; ^‡^All values presented in mg/dl; ^§^p-value from a χ^2^ test and ^||^p-value form a test for trend.

### Association of seven SNPs with various lipid traits

Out of the seven SNPs which passed quality control, two variants, rs562338 (*APOB*) and rs6511720 (*LDLR*)) were previously demonstrated to be associated with LDL-cholesterol levels; two SNPs, rs1864163 (*CETP*) and rs4775041 (*LIPC*)) as associated with HDL-cholesterol levels and three polymorphisms rs780094 (*GCKR*), rs10503669 (*LPL*) and rs662799 (*APOA5*) as associated with triglyceride levels in European populations [4], [9,10]. In our analysis, we found corresponding associations for two SNPs with HDL and LDL-cholesterol levels. We replicated one (*APOB*) of the two tested associations with LDL-cholesterol as a primary trait (Table 2). Of the two primary trait associations tested with HDL-cholesterol at the *CETP* and *LIPC* loci, we replicated the genetic effect at the *LIPC* locus. We replicated all three tested primary trait associations with triglyceride levels. The p-values for these associations ranged from 0.03 to 1.97 x 10^-7^ and the proportion of variation explained by these SNPs ranged from 0.001 to 0.01. Briefly, we identified that the risk allele in rs562338 at *APOB* locus predicted 4.7 mg/dl lower LDL-cholesterol levels (95% CI: 0.52, 8.9 mg/dl, p=0.03), rs4775041 at *LIPC* locus associated with 2.6 mg/dl higher HDL-cholesterol levels (95% CI: 0.19, 1.33 mg/dl, p=0.007), rs780094 at *GCKR* altered higher triglyceride levels by 7.6 mg/dl (95% CI: 3.4, 12.4 mg/dl, p=0.001), rs10503669 at *LPL* locus was marked by 12.4 mg/dl lower triglyceride levels (95% CI: 6.2, 17.9 mg/dl, p=1.0 x 10^-4^) and rs662799 at *APOA5* is associated with 12.4 mg/dl higher triglyceride levels (95% CI: 7.6, 17.3 mg/dl, p=1.97 x 10^-7^).

**Table 2:** Within sib pair association statistics for seven SNPs with various lipid traits

**Gene**	**Reference for test SNP**	**Effect Size from European GWAS**	**SNP (Minor allele frequency)**	**Phenotype associated with test SNP in previous GWAS**	**Triglyceride**	**Total Cholesterol**	**HDL**-**cholesterol**	**LDL**-**cholesterol**
*APOA5*	[10]	16.88 mg/dl [10]	rs662799 (G=0.19)	Triglyceride	0.18 (0.11, 0.25, **p**=**1**.**97 x 10**^-**7**^)	0.08 (0.02, 0.14, **p**=**0**.**01**)	−0.04 (−0.10, 0.02, p=0.21)	0.05 (−0.01, 0.12, p=0.12)
*APOB*	[9]	4.89 mg/dl [9]	rs562338 (A=0.13)	LDL-Cholesterol	−0.006 (−0.09, 0.07, p=0.87)	−0.08 (−0.15, -0.002, **p**=**0**.**04**)	0.03 (−0.04, 0.10, p=0.44)	−0.09 (−0.17, -0.01, **p**=**0**.**03**)
*CETP*	[10]	4.12 mg/dl [10]	rs1864163 (A=0.20)	HDL-Cholesterol	−0.004 (−0.07, 0.07 , p=0.90)	−0.05 (−0.12, 0.01, p=0.12)	−0.01 (−0.08, 0.05, p=0.65)	−0.05 (−0.11, 0.02, p=0.18)
*GCKR*	[10]	8.59 mg/dl [10]	rs780094 (T=0.21)	Triglyceride	0.11 (0.05, 0.18, **p**=**0**.**001**)	0.11 (0.05, 0.17, **p**=**3**.**9x10**^-**4**^)	0.04 (−0.01, 0.10, p=0.15)	0.07 (0.004, 0.13, **p**=**0**.**03**)
*LDLR*	[4]	−6.99 mg/d [4]	rs6511720 (T=0.07)	LDL-Cholesterol	0.005 (−0.11, 0.012, p=0.93)	−0.06 (−0.17, 0.05, p=0.28)	0.04 (−0.06, 0.15, p=0.39)	−0.06 (−0.18, 0.04, p=0.24)
*LIPC*	[10]	1.38 mg/dl [10]	rs4775041 (C=0.21)	HDL-Cholesterol	0.03 (−0.04, 0.10, p=0.40)	0.006 (−0.05, 0.07, p=0.84)	0.08 (0.02, 0.14, **p**=**0**.**007**)	−0.03 (−0.09, 0.03, p=0.35)
*LPL*	[10]	2.09 mg/dl [10]	rs10503669 (A=0.12)	Triglyceride	−0.18 (−0.26, -0.09, **p**=**1**.**0 x 10**^-**4**^)	−0.09 (−0.17, 0.007, **p**=**0**.**03**)	0.03 (−0.05, 0.11, p=0.42)	−0.04 (−0.12, 0.04, p=0.36)

Data presented as β specific for z-scores with 95% CI and p-value.

### Association tests for SNPs at seven loci with secondary lipid measures

Apart from association of seven SNPs with the lead lipid traits identified in the European populations, we also analyzed their influence on secondary lipid traits. Unlike association tests with lead traits, this exploratory analysis was more susceptible to type-1 error given the greater number of null hypotheses, which were tested. In our screen for secondary associations, we identified five associations at p≤0.05 (Table 2). Based on a Bonferroni correction for 21 tests (p≤0.002), a variant at the *GCKR* locus (rs780094) was associated with higher total cholesterol levels (beta=0.11 [0.05, 0.17], p=3.9x10^-4^), which remained even after adjusting for body mass index (BMI), hypertension and fasting glucose levels (β=0.11, p=3.5 x 10^-4^).

### Association of lipid associated SNPs with secondary cardiovascular risk factor measures

In Table 3, we describe results from an association screen of seven SNPs with hypertension, systolic blood pressure, diastolic blood pressure, fasting glucose, fasting insulin and BMI which are established risk factors for cardiovascular diseases. We identified two associations with hypertension, of which the strongest was at the *LDLR* locus with individuals carrying the minor allele of the variant rs6511720 having higher risk of hypertension (OR=1.88 [1.23 - 2.88], p=0.003). This SNP was not observed to be associated with any of the cardiovascular traits. Including the lipid traits or obesity (BMI) as covariates did not influence the strength of association. The minor allele at the *LPL* locus was associated with lower triglyceride and total cholesterol levels (Table 2) and was further observed to lower systolic blood pressure, fasting glucose levels and fasting insulin levels (Table 3). The *GCKR* variant associated with multiple lipid phenotypes was also observed to increase the risk of hypertension (OR=1.26 [1.00, 1.59] p= 0.05). None of the associations with secondary traits survived the Bonferroni correction based on the number of tests reported in Table 3 (p_corr_=0.002). Apart from lipid traits previous results from European studies have established the rs6511720 variant at the *LDLR* locus as also associated with carotid intima media thickness, plaque and CAD [11]. Other associations which need to be followed up in future studies include the *LPL* and *GCKR* loci. Although the associations of the *LPL* variant with systolic blood pressure, fasting glucose and fasting insulin and that of the *GCKR* variant with hypertension did not remain statistically robust following multiple hypothesis test correction, each of these loci were associated with multiple traits in an independent manner suggesting pleiotropic effects which can only be established by further studies. The studied variant at the LPL locus was also associated total cholesterol levels, however an association with HDL-Cholesterol as observed in the original European GWAS and meta-analysis was not seen [10]. The GCKR variant demonstrated strong associations with total-cholesterol and triglyceride levels and nominal associations with hypertension and LDL-Cholesterol, however previous reports of associations with fasting glucose levels [12] were not confirmed in this study.

**Table 3:** Association of lipid associated SNPs with hypertension and diabetes related traits based on minor allele and within sib pair association estimates from the Fulker model

**Gene**	**SNP**	**Hypertension**	**Systolic blood pressure**	**Diastolic blood pressure**	**Fasting glucose**	**Fasting insulin**	**BMI**
*APOA5*	rs662799	0.84(0.65, 1.08), 0.18	−0.02(−0.09, 0.04), 0.51	−0.002(−0.07, 0.06), 0.12	−0.014(−0.08, 0.05), 0.67	0.04(−0.02, 0.11), 0.20	0.014(−0.05, 0.08), 0.63
*APOB*	rs562338	0.79(0.58, 1.06), 0.12	−0.05(−0.13, 0.02), 0.17	−0.03(−0.11, -0.05), 0.45	−0.02(−0.10, 0.05), 0.53	0.01(−0.07, 0.09), 0.74	0.02(−0.05, 0.10), 0.49
*CETP*	rs1864163	1.02(0.79, 1.31), 0.85	−0.03(−0.09, 0.03), 0.36	−0.03(−0.10, 0.03), 0.35	0.02(−0.04, 0.09), 0.49	0.009(−0.06, 0.08), 0.81	0.03(−0.03, 0.09), 0.40
*GCKR*	rs780094	1.26(1.00, 1.59), **0**.**05**	0.02(−0.04, 0.08), 0.46	0.04(−0.02, 0.10), 0.22	−0.01(−0.08, 0.05), 0.63	0.06( −0.003, 0.13), 0.06	−0.004(−0.06, 0.05), 0.88
*LDLR*	rs6511720	1.88(1.23, 2.88), **0**.**003**	0.06(−0.05, 0.17), 0.28	−0.01(−0.12, 0.10), 0.82	0.02(−0.09, 0.13), 0.73	−0.08(−0.19, 0.04), 0.19	−0.01(−0.11, 0.09), 0.84
*LIPC*	rs4775041	0.98(0.78, 1.22), 0.83	−0.004(−0.07, 0.06), 0.9	−0.01(−0.08, 0.05), 0.65	−0.009(−0.07, 0.05), 0.79	0.03(−0.04, 0.09), 0.43	−0.02(−0.08, 0.03), 0.42
*LPL*	rs10503669	0.74(0.54, 1.02), 0.07	−0.09(−0.17, -0.005), **0**.**04**	−0.04(−0.13, 0.04), 0.35	−0.10(−0.18, -0.01), **0**.**02**	−0.11(−0.19, -0.02), **0**.**02**	−0.04(−0.12, 0.03), 0.28

### Comparison of linkage disequilibrium patterns between Indian and European HapMap samples

Extreme variation in LD correlations across European and Indian populations can lead to a dilution of genetic effects due to a breakdown in LD correlation structure. Using HapMap-CEPH and HapMap-GIH data we did not observe large differences in LD correlation matrices at the seven loci between the two populations after correction for multiple tests (Table 4). The smallest empirical p-value was observed at the *APOA5* locus (p=0.04). Thus, replication of five of the seven lipid SNP effects could be due to conservation of linkage disequilibrium structure across these loci, and that the non-replication of the remaining two SNPs cannot be explained by systematic differences in LD, although it remains possible that the pair wise LD between the SNPs studied and the unknown causal variant may differ between populations.

**Table 4:** **Summary of Zaykin**’**s z**_**2 **_**statistic based on LD correlation structures in HapMap CEU and GIH populations**

**SNP (Gene)**	**Number of SNPs in the LD comparison matrix**	**Number of z**_**2 **_**values in random permutations greater than observed z**_**2 **_**value**	**Number of z**_**2 **_**values in random permutations lower than observed z**_**2 **_**value**	**Empirical p**-**value (one**-**sided test for difference in LD)**
rs662799 (*APOA5*)	25	4302	95699	**0**.**04**
rs562338 (*APOB*)	14	26258	73743	0.26
rs1864163 (*CETP*)	30	23107	76894	0.23
rs780094 (*GCKR*)	13	27258	72743	0.27
rs6511720 (*LDLR*)	6	17146	82855	0.17
rs4775041 (*LIPC*)	40	41804	58917	0.41
rs10503669 (*LPL*)	36	36613	63388	0.37

### Analysis of demographic and dietary factors as potential effect modifiers

We tested for effect modification by sex, dietary fat intake and location in relation to lipid trait levels while allowing for main effects of the five SNPs which were associated with lipid traits. Results for this analysis are presented in Table 5, 6 and 7. None of five tested SNPs had any modifying effect on the genetic effect on any lipid trait. The only gene-environment interaction term that was of interest was at the *LDLR* where the effect of *LDLR* locus on LDL-Cholesterol was dependent on sex (β=0.27 [0.01, 0.52], p=0.04). However, the present study was underpowered to detect modest gene-environment interaction effect sizes and this finding is likely due to the play of chance. To further explore differences in effect sizes, we performed stratified analysis to estimate SNP effect sizes based on sex, dietary fat intake and location. Here we observed variation in regression coefficients between males and females in relation to the strength of primary trait associations at the *APOB*, *LDLR* and *LIPC* loci (Table 5). Similarly fluctuations in regression coefficients were observed with respect to prediction of HDL-Cholesterol levels between urban 0.05 (−0.04, 0.13, p=0.29) and rural locations 0.17 (0.04, 0.29, p=0.007) by the *LIPC* locus variant, and the prediction of LDL-cholesterol levels between participants accustomed to low fat 0.02 (−0.15, 0.20, p=0.77) or high fat diet −0.20 (−0.36, -0.04, p=0.02) by the *LDLR* locus variant (Table 7). Given that these associations are not corrected for multiple hypotheses testing, it is likely that they are chance findings and require replication in well-powered South Asian studies.

**Table 5:** Within sib pair association statistics for seven SNPs with lead lipid traits after stratifying for Sex

**Gene**	**Trait tested**	**Males**	**Females**	**Interaction effect**
*APOA5*	Triglycerides	0.19 (0.09, 0.29, p=**0**.**0002**)	0.17 (0.07, 0.27, p=**0**.**001**)	0.10 (−0.04, 0.25, p=0.16)
*APOB*	LDL-Cholesterol	−0.01 (−0.13, 0.10, p=0.79)	−0.19 (−0.32, -0.06, p=**0**.**005**)	−0.17 (−0.35, 0.01), p=0.07
*CETP*	HDL-Cholesterol	−0.02 (−0.11, 0.07, p=0.63)	0.01 (−0.09, 0.12, p=0.79)	0.03 (−0.11, 0.18, p=0.66)
*GCKR*	Triglycerides	0.09 (−0.006, 0.18, p=0.07)	0.13 (0.03, 0.23, p=**0**.**009**)	0.06 (−0.09, 0.21, p=0.42)
*LDLR*	LDL-Cholesterol	−0.18 (−0.33, -0.02, p=**0**.**02**)	0.10 (−0.09, 0.28, p=0.30)	0.27 (0.01, 0.52, p=**0**.**04**)
*LIPC*	HDL-Cholesterol	0.04 (−0.04, 0.12, p=0.36)	0.13 (0.02, 0.23, p=**0**.**01**)	0.09 (−0.05, 0.23, p=0.21)
*LPL*	Triglycerides	−0.14 (−0.27, -0.01, p=**0**.**03**)	−0.21 (−0.34, -0.08, p=**0**.**001**)	−0.06 (−0.26, 0.13, p=0.54)

Data presented as β specific for z-scores with 95% CI and p-value.

**Table 6:** Within sib pair association statistics for seven SNPs with lead lipid traits after stratifying for location

**Gene**	**Trait tested**	**Urban**	**Rural**	**Interaction effect**
*APOA5*	Triglycerides	0.18 (0.09, 0.27, p=**0**.**0001**)	0.19 (0.06, 0.32, p=**0**.**004**)	−0.009 (−0.18, 0.16, p=0.91)
*APOB*	LDL-Cholesterol	−0.04 (−0.20, 0.12, p=0.63)	−0.11 (−0.22, -0.005, p=0.34	−0.06 (−0.26, 0.13, p=0.53)
*CETP*	HDL-Cholesterol	−0.02 (−0.16, 0.22, p=0.78)	−0.005 (−0.09, 0.08, p=0.90)	0.04 (−0.13, 0.21, p=0.64)
*GCKR*	Triglycerides	0.11 (0.03, 0.20, p=**0**.**009**)	0.14 (0.01, 0.26, p=**0**.**03**)	−0.004 (−0.16, 0.15, p=0.96)
*LDLR*	LDL-Cholesterol	−0.05 (−0.20, 0.09, p=0.47)	−0.13 (−0.36, 0.10, p=0.26)	0.04 (−0.13, 0.21, p=0.64)
*LIPC*	HDL-Cholesterol	0.05 (−0.04, 0.13, p=0.29)	0.17 (0.04, 0.29, p=**0**.**007**)	−0.11 (−0.27, 0.05, p=0.18)
*LPL*	Triglycerides	−0.21 (−0.32, -0.09, p=**0**.**001**)	−0.14 (−0.30, 0.02, p=0.09)	−0.07 (−0.28, 0.14, p=0.50)

Data presented as β specific for z-scores with 95% CI and p-value.

**Table 7:** Within sib pair association statistics for seven SNPs with lead lipid traits after stratifying for fat intake

**Gene**	**Trait tested**	**Low fat intake**	**Medium fat intake**	**Interaction effect**
*APOA5*	Triglycerides	0.18 (0.08, 0.29, p=**3**.**4 x 10**^-**4**)^	0.19 (0.08, 0.29, p=**0**.**001**)	0.04 (−0.10, 0.19, p=0.55)
*APOB*	LDL-Cholesterol	−0.11 (−0.23, 0.02, p=0.09)	−0.07 (−0.18, 0.04, p=0.21)	0.06 (−0.12, 0.23, p=0.53)
*CETP*	HDL-Cholesterol	0.01 (−0.08, 0.11, p=0.78)	−0.03 (−0.13, 0.07, p=0.61)	−0.03 (−0.18, 0.11, p=0.63)
*GCKR*	Triglycerides	0.11 (0.02, 0.21, p=**0**.**02**)	0.13 (0.03, 0.23, p=**0**.**01**)	−0.03 (−0.17, 0.11, p=0.69)
*LDLR*	LDL-Cholesterol	0.02 (−0.15, 0.20, p=0.77)	−0.20 (−0.36, -0.04, p=**0**.**02**)	−0.03 (−0.18, 0.11, p=0.63)
*LIPC*	HDL-Cholesterol	0.09 (−0.004, 0.18, p=0.06)	0.09 (−0.004, 0.19, p=0.06)	0.05 (−0.09, 0.19, p=0.48)
*LPL*	Triglycerides	−0.14 (−0.27, -0.004, p=**0**.**04**)	−0.21 (−0.34, -0.07, p=**0**.**002**)	−0.01 (−0.20, 0.17, p=0.87)

Data presented as β specific for z-scores with 95% CI and p-value.

## Discussion

We aimed to replicate association of eight previously identified lipid associated loci. After quality control checks, we tested for associations of seven SNPs in distinct loci with lipid traits. We identified associations for five of the seven tested SNP associations and found the direction of association for the two remaining SNPs to be consistent with previous findings in the European population.

As variation in key lipid traits can be a result of genetic variants and lifestyle related factors, it is important to identify and characterize the proportion of variation that can be attributed to genetic factors alone. Few genetic association studies in relation to lipid traits in South Asian populations have been published recently. Kooner et al. [13] performed a GWAS in 1,006 Indian Asians from the LOLIPOP study and identified variants in the *APOA1-APOC3-APOA4-APOA5* and the *MLXIPL* as associated with triglyceride levels. Two further studies attempted replication of European GWAS findings using the same cohort of 9000 south Asian individuals [4,14]. While Teslovich et al. identified majority of association signals in South Asians as being in the same direction as in European population (4), Kooner et al. replicated 3 out of 8 associations they had initially identified in their primary GWAS analysis among individuals of European ancestry. Another study in a South Indian population identified variants in the *LPL* gene to be associated with HDL-cholesterol and hypertriglyceridemia [15].

The strongest finding in our association screen for primary traits was observed at the APOA5 locus where the minor allele at the rs662799 variant was associated with higher triglyceride levels. This is consistent with earlier studies on Indian subjects from Pune and New Delhi [16,17]. The effect size observed in this study was equivalent to an increase in triglyceride levels of 12.4 mg/dl (per allele effect size= 0.18 s.d units (0.11, 0.25)) per minor allele at the rs662799 variant which is lower than the predicted 16.88 mg/dl increase as published by Willer et al. [10] for the same SNP in their South Asian samples.

The rs780094 variant in the *GCKR* predicted higher triglyceride and total cholesterol levels. The association of *GCKR* locus with total cholesterol levels was the only secondary trait association that remained associated after Bonferroni correction. This variant has previously been described to be in strong LD with a non-synonymous variant rs1260326 (Pro446Leu) [12] (r^2^=0.89 and 0.96 in HapMap-CEPH and HapMap-GIH samples respectively).

Further associations which were replicated included a SNP (rs4775041), 49 kb upstream of the *LIPC* gene and the major “C” allele at this variant has previously been shown to be associated with 1.38 mg/dl increase in HDL-C levels [10]. In contrast, we observed the “C” allele as the minor allele that predicted an increase in HDL-C levels equivalent to 0.76 mg/dl ((per allele effect size= 0.08 s.d units (0.02, 0.14)) per copy of the “C”-allele. The rs10503669 variant at the *LPL* locus was previously shown to be associated with 11.57 mg/dl increase in triglyceride levels per copy of the common “C” allele [10]. In addition, this variant was also associated with HDL-cholesterol levels. We observed the minor “A” allele at this variant to be associated with 12.44 mg/dl (per allele effect size= −0.18 s.d units (−0.26, -0.09)) lower triglyceride levels and 4.06 mg/dl (per allele effect size= −0.09 s.d units (−0.17, 0.0007)) lower total cholesterol levels.

The genotyped variant at the *LDLR* locus (rs6511720) is strongly associated with cholesterol levels [18,4] in European populations, but we could not identify association of this locus with either total cholesterol or LDL and HDL- cholesterol levels. This could be because of insufficient statistical power to detect a potentially weaker effect in Indian population. Variants at the *LDLR* gene were not studied in South Asian samples from the LOLIPOP study as part of the Global lipid genetics consortium hence comparable results are not available ^4^. Future studies in South Asian populations will need to explore whether a different variant and haplotype than the one observed in Europeans is associated with LDL-Cholesterol levels. The other genetic association we failed to replicate was observed at the *CETP* locus. Although a rcent replication study (~20,000) which followed the Global lipid genetics consortium project too failed to replicate the association of rs1864163 variant at *CETP* with HDL-cholesterol in European Americans [19], insufficient sample size in our analysis is the likely explanation for the non-replication at this locus.

In an exploratory analysis, we studied whether the seven SNPs included in the association screen for primary lipid traits were also associated with hypertension and blood pressure. We identified an association between *LDLR* variant and increased risk of hypertension, which was not influenced by adjusting for lipid traits and obesity parameters. The same variant at the *LDLR* locus has previously been shown to be associated with myocardial infarction and Apo B/A1 ratio [20]. Further it was shown that adjusting for Apo B/A1 ratio rendered the association with myocardial infarction statistically null. The rs10503669 variant at the *LPL* locus, was observed to be associated with total cholesterol levels and systolic blood pressure. A different variant rs12678919 in complete LD with rs10503669 (r^2^=1) was found to be associated with triglyceride and cholesterol levels in the south Asian samples in the LOLIPOP study. In this study, we have replicated these genetic effects at the *LPL* locus. The statistical evidence is lacking as neither of these two secondary associations survive a correction for multiple testing. Further investigations in larger sample sizes in the south Asian populations should be encouraged given the prior evidence from GWAS studies at the *LDLR* locus and the biological candidacy of the *LPL* locus.

Associations with primary lipid traits identified in this study include strong biological candidates with convincing statistical evidence for association from previous studies. *APOA5* and *APOB* are apolipoproteins involved in transport of lipids; *LPL* and *LIPC* are lipases and *GCKR* actively manipulates glucose and triglyceride concentrations through its role in the GCKR-pathway [12]. Future studies will need to explore several common variants which have subsequently been identified to be associated with blood lipid traits [4]. In conclusion, we have successfully replicated five of the seven established associations between SNPs and lipid traits in the largest replication study in an indigenous Indian population sample. The similarity of LD correlation structures across the European and Indian populations supports our results and suggests conservation of genetic roles across ethnicity and varying environmental conditions.

## Methods

### Study population

Phenotypic information was available on 7068 participants from the Indian Migration Study, of whom 6774 individuals were full sibling pairs. Genetic and phenotypic information from 3178 sib-pairs was used for association analysis. IMS was conducted as part of a Cardiovascular Disease Risk Factor Screening framework and participants were recruited from industrial areas in 4 different cities (Lucknow, Nagpur, Bangalore and Hyderabad) [21]. Factory workers and their co-resident spouses were recruited if they were rural–urban migrants. Each migrant worker and spouse was asked to invite one non-migrant full sibling of the same sex and closest to them in age and still residing in their rural place of origin [22]. Ethical approval was obtained from the ethics committee of the All India Institute of Medical Sciences, New Delhi, India (AIIMS; reference number A-60/4/8/2004).

### Biochemical phenotypes

After the separation of plasma & serum, samples were transported monthly to AIIMS, New Delhi, for biochemical assays. Serum HDL-Cholesterol was estimated directly by an elimination method [23], total cholesterol by an enzymatic endpoint method, and triglycerides by GPO-PAP method using kits from Randox Laboratory Ltd. (Crumlin City, United Kingdom). Low density lipoprotein cholesterol level was estimated using the Friedewald-Fredrickson formula [24]. The quality of local assays was cross-checked with regular external standards and internal duplicate assays and monitored by AIIMS. For quality assurance the Cardiac Biochemistry Lab, AIIMS, is part of the UK National External Quality Assessment (http://www.ukneqas.org.uk/). Hypertension was defined as either a systolic blood pressure ≥140 mmHg or a diastolic blood pressure ≥90 mmHg [25]. Fasting plasma glucose was measured on the day of blood collection by local laboratories at each site using the GOD-PAP method and RANDOX kits (Randox Laboratories, Crumlin, UK) [26]. Fasting insulin was assayed in serum samples by the ELISA method, as a solid-phase two-site enzyme immunoassay, using kits from MERCODIA (Mercodia AB, Sylveniusgatan, Uppsala, Sweden) [27]. Body mass index was calculated as weight in kilograms divided by the squared product of height measured in metres.

### SNP selection and genotyping

The eight lipid associated SNPs were genotyped in parallel with 51 other SNPs reported to be associated with type-2 diabetes, obesity, myocardial infarction and height at the time of inception of this study in 2008. The choice of SNPs was restricted to those variants which were associated with lipid trait/traits at genome wide levels of significance in European studies and were also biologically plausible given their location in genes known to be linked with Mendelian abnormalities of lipid metabolism. The selected loci are strong biological candidates given that rare mutations in seven of them (excluding *GCKR*) have been observed in Mendelian abnormalities of lipid metabolism. These abnormalities include hypertriglyceridemia [28,29], familial hypercholesterolemia [30,31], complete hepatic lipase (HL) deficiency [32], type I hyperlipidemia [33] and Familial dysbetalipoproteinemia [34]. Hence a further aim of this study was to assess the role of common variants in genes which are linked with rare forms of Mendelian abnormalities of lipid metabolism in regulating lipid levels on a population level. We included the *GCKR* locus in the Genomic DNA samples already stored in 96 deep well storage plates at a uniform concentration of 10ng/λ were used for genotyping using sequenom based Mass ARRAY assay technology. For quality control purposes, ~10% (n=8) duplicates were incorporated into each of the 96 deep well storage plates.

### Statistical analysis

#### SNP quality control and association models

Deviation from Hardy-Weinberg equilibrium (HWE) was tested using the exact test implemented in Plink [35] for all the 8 SNPs in unrelated IMS participants (N=3387), while employing both overall IMS samples and city-wise samples. Any SNP that failed HWE test (P-value ≤0.001) in participants from any of the 4 cities or the overall IMS sample was excluded from association analysis. We have previously reported evidence of population substructure within IMS with potentially up to 10 population subgroups [36].

Association analysis was performed using Fulker’s genetic association model which decomposes genotypes into between and within sib-pair association effects [37]. Both these effects were modeled independently and inferences were drawn based on the within sib-pair component which is unaffected by population substructure. Linear mixed effect regression models were used for association analysis. The main effect model included the lipid trait as the dependent variable and between and within sib-pair coding of the test SNP as explanatory variables. The lipid trait marked as the lead trait for each SNP was based on its association at genome wide significance in previous GWASs and subsequent replication in independent studies. Lipid phenotypes other than the lead traits were described as secondary traits. Standardized z-scores of lead and secondary traits were used as the dependent variables.

Covariates included age, sex, location (urban or rural) and city. A random sib-pair effect was included to allow for shared environmental and polygenic effects. Association analysis using mixed effect models was implemented using STATA v11.2 (Stata corp, Texas, USA) and UNPHASED [38]. Percentage of variance explained by the test SNPs was measured by calculating the additive genetic variation for each of the test SNP and dividing this value by the total phenotypic variance for the lipid trait being studied. Additive genetic variance was estimated as Va= 2pq β^2^, where β is the regression coefficient of the within sib-pair component of Fulker’s genetic association model and p and q are the allele frequencies for the major and minor alleles. This estimate of additive genetic variation is based on the classical formula used for non-familial genotype data where Va = (2pq[p(X_11_ − X_12_) + q(X_12_–X_22_)]^2^) [39] and X_11,_ X_12_ and X_22_ are estimated means of lipid traits for common allele homozygotes, heterozygotes and rare allele homozygotes respectively.

For stratified analysis dietary fat intake was defined by a binary variable which grouped individuals as low and high fat intake after defining the median as the cut-off point. Fat intake was assessed by an interviewer-administered semi-quantitative food frequency questionnaire (FFQ). Gene-Environment (Diet, Location and Sex) interaction effects were tested while including Gene-Environment interaction terms within the fixed effects component of Fulker’s association model.

### Comparison of linkage disequilibrium (LD) correlation patterns for lipid trait genes between HapMap-GIH and CEPH

We used genotype data available from phase-3 of HapMap-CEPH (Centre d'Etude du Polymorphisme Humain) and GIH (Gujarati Indians in Houston) populations to compare the linkage disequilibrium between the two population groups. The overall difference in pair wise LD (r^2^) was calculated using the z_2_ statistic of Zaykin et al. [40], which is the sum of squared element-wise differences between two LD matrices [40]. We performed 100,000 permutations of subject ethnicity to assess the significance of the z_2_ statistic under the null hypothesis that the two populations have the same average pair-wise LD. Permutation tests were performed using the cvpermute command in MATA in STATA v11.2 (Stata corp, Texas, USA).

### Power calculations

Given a minor allele frequency (MAF) range of 7-21% and a sample size of 3178 sib pairs; we had over ≥80% power to detect associations which explained ≥0.003% of variation in the studied lipid traits. Sample size calculations were performed using the Genetic Power Calculator [41] at statistical significance of p≤0.05 since this was a replication study. For stratified analysis in females (42.3%), in participants from rural location (36.6%) and in individuals on low dietary fat intake (50%), we had ≥80% power to detect effect sizes which explained ≥0.006%, ≥0.007% and ≥0.005% of variation in quantitative traits.

## Abbreviations

LD: Linkage Disequilibrium; SNP: Single nucleotide polymorphism; APOA5: Apolipoprotein A-V gene; APOB: Apolipoprotein B gene; APOE/CI/C4: Apolipoprotein *E*/*C-I*/C-IV gene cluster; CETP: Cholesteryl ester transfer protein gene; LDLR: Low density lipoprotein receptor gene; GCKR: Glucokinase (hexokinase 4) regulator gene; LPL: Lipoprotein lipase gene; SNP: Single nucleotide polymorphism; GWAS: Genome wide association study.

## Competing interests

The authors declare that they have no competing interests.

## Author contributions

SE, GDS, FD, DP, YB-S, KSR, GRC, SK and IMSG contributed to the conception and design of the study. SR, KKV, VG, CJS, SP, SNM, SK, NJT, GDS, FD, YB-S, SE and GRC contributed to the analysis and interpretation of data. SR, FD, SE, GRC and GDS were responsible for drafting the article. SR, VG, FD, SE, GRC, YB-S, DP, VG, SK, LB and NJT contributed to critical revision of the paper. All authors have read the manuscript and gave their final approval for this version of the manuscript to be published.
